# Efficacy of Indirect Decompression by Posterior Longitudinal Ligament Ligamentotaxis in Minimally Invasive Oblique Lateral Interbody Fusion (MIS-OLIF) Without Posterior Decompression for Degenerative Lumbar Disease

**DOI:** 10.3390/jcm15176572

**Published:** 2026-08-26

**Authors:** Jung-Woo Hur, Dong Hun Kim, Seonyong Yun, Jae Taek Hong

**Affiliations:** 1 Department of Neurosurgery, Eunpyeong St. Mary’s Hospital, College of Medicine, The Catholic University of Korea, Seoul 03312, Republic of Korea; 2 Department of Neurosurgery, Bucheon St. Mary’s Hospital, College of Medicine, The Catholic University of Korea, Bucheon 14647, Republic of Korea

**Keywords:** oblique lateral interbody fusion, OLIF, indirect decompression, ligamentotaxis, posterior longitudinal ligament, lumbar spinal stenosis, spondylolisthesis, minimally invasive spine surgery

## Abstract

**Background/Objectives:** Indirect decompression through restoration of disc and foraminal height with a large lateral interbody cage has been advocated for selected patients, but its efficacy in a large series remains unproven. In this retrospective study, we evaluated the clinical and radiographic results of indirect decompression achieved by posterior longitudinal ligament (PLL) ligamentotaxis in minimally invasive oblique lateral interbody fusion (MIS-OLIF) performed without posterior decompression. **Methods:** We retrospectively reviewed 236 consecutive patients treated for single- or two-level degenerative lumbar disease (November 2019–May 2025); clinical follow-up of at least 24 months was available in 181 patients. Visual analogue scale (VAS) scores and radiographic parameters—disc height, foraminal height, foraminal area, spinal canal diameter and cross-sectional area (CSA) of the thecal sac—were compared before and after surgery, and extension ratios were correlated with preoperative values. **Results:** Clinical scores improved significantly. All radiographic parameters increased substantially: disc height +49.1%, foraminal height +33.7%, foraminal area +44.5%, canal diameter +37.4% and CSA +36.2%. Extension ratios were inversely correlated with preoperative values. Six patients (2.5%) required additional posterior decompression. The slippage subgroup showed greater radiographic gains than the stenosis subgroup. **Conclusions:** MIS-OLIF without posterior decompression significantly enlarged the foramen and spinal canal; reduction in disc bulging and PLL ligamentotaxis may contribute to this effect, with greater improvement in more severely degenerated, overtly slipped segments. Careful patient selection remains essential, and prospective comparative studies with longer follow-up are needed.

## 1. Introduction

Degenerative lumbar disease—comprising spinal stenosis, degenerative spondylolisthesis and herniated intervertebral disc—is a leading cause of low back pain, radiculopathy and neurogenic claudication, and its prevalence continues to rise with the aging population. When conservative management fails, surgical decompression of the symptomatic neural elements remains the mainstay of treatment, frequently combined with interbody fusion to address segmental instability and to restore sagittal balance.

Decompression can be achieved either directly or indirectly. Direct decompression—laminectomy, facetectomy, foraminotomy or discectomy—removes the impinging bone, ligament or disc material and provides immediate enlargement of the neural space. However, open posterior decompression entails recognised risks, including incidental durotomy, nerve root injury, epidural haematoma, postoperative epidural fibrosis and iatrogenic instability arising from resection of the posterior osteoligamentous complex. Indirect decompression instead enlarges the spinal canal and neural foramen without direct manipulation of the neural elements, thereby preserving the posterior structures and avoiding many of these complications.

Indirect decompression techniques can be classified as segmental procedures, which act at a single motion segment, or global alignment procedures, which restore decompression through correction of overall spinal alignment [1]. Among the segmental procedures, lateral access interbody fusion has attracted particular attention. By inserting a large-footprint cage across the apophyseal ring, these techniques distract the disc space, restore disc and foraminal height, tension the annulus fibrosus and the posterior longitudinal ligament (PLL), and reduce buckling of the ligamentum flavum, collectively expanding the central canal and the neural foramina. Radiographic and clinical evidence of effective indirect decompression after extreme lateral and oblique lateral interbody fusion has been reported [2,3].

The concept that tensioning of the PLL can reduce retropulsed material into the disc space—ligamentotaxis—was first established in the management of thoracolumbar burst fractures, in which distraction and PLL tension help restore the canal contour. The same biomechanical principle is thought to operate during interbody distraction in degenerative disease: as the disc space is re-expanded, the taut PLL and annulus pull the bulging disc and the posteriorly displaced tissues anteriorly, away from the dural sac and the exiting nerve roots.

Oblique lateral interbody fusion (OLIF) employs a pre-psoas, retroperitoneal corridor anterior to the psoas muscle and lumbar plexus. Anatomically, this window exploits the natural interval between the great vessels anteromedially and the psoas major with the lumbar plexus posterolaterally, so that the disc space can be reached without dividing the posterior paraspinal musculature, the facet joints or the posterior tension band; a minimally invasive tubular corridor further limits iatrogenic muscle denervation and soft-tissue disruption. This anatomy is the rationale for a posterior-sparing, minimally invasive approach: the compressive burden is relieved indirectly from the front, while the stabilising posterior column is left intact [4,5]. Compared with the trans-psoas direct lateral approach (DLIF/XLIF), the oblique corridor reduces the risk of lumbar plexus and psoas injury, lessens the dependence on intraoperative neuromonitoring, and improves access to the L4–5 level, which is often obscured by a high-riding iliac crest in the lateral approach [4,5]. OLIF combined with posterior percutaneous fixation but without posterior decompression has been reported to indirectly decompress the spinal canal in degenerative spondylolisthesis [6].

Despite the growing enthusiasm for indirect decompression with large lateral cages, its efficacy has not been demonstrated in a large consecutive patient series, and concern persists regarding its adequacy for central stenosis and bony lateral recess compromise. Most existing OLIF series report modest cohorts, frequently combine the procedure with planned posterior decompression, and seldom relate the magnitude of decompression to the preoperative severity of the segment. Consequently, the selection of candidates for stand-alone indirect decompression—in particular, the distinction between segments that will respond to ligamentotaxis and those with fixed bony compression that will not—remains poorly defined. The present study addresses these gaps in a large, consecutive, single-protocol cohort treated strictly without any posterior decompression.

The purpose of the present study was to demonstrate the clinical and, in particular, the radiographic results of indirect decompression achieved by PLL ligamentotaxis in MIS-OLIF performed without any posterior decompression, and to identify the preoperative factors associated with the magnitude of the decompressive effect. We hypothesised that (i) MIS-OLIF without posterior decompression produces significant enlargement of the neural foramen and the central canal; (ii) the magnitude of indirect decompression is inversely related to the preoperative dimensions of each segment, so that more severely collapsed or stenotic segments improve proportionally more; and (iii) segments with overt spondylolisthesis achieve greater radiographic gains than stenosis-only segments.

## 2. Materials and Methods

### 2.1. Study Design and Patients

This was a retrospective, single-institution review of prospectively collected data. We identified 236 consecutive patients who underwent MIS-OLIF without posterior decompression for single- or two-level degenerative lumbar disease between the L1 and L5 vertebrae from November 2019 to May 2025 (66 months). Eligible diagnoses were lumbar spinal stenosis, degenerative or low-grade isthmic spondylolisthesis, and herniated intervertebral disc with corresponding neurogenic claudication or radiculopathy refractory to conservative care. Patients requiring planned posterior decompression, those with high-grade (>grade II) spondylolisthesis, gross instability mandating extensive open fusion, infection, tumour or fracture, and those with inadequate radiographic follow-up were excluded. The study was conducted in accordance with the Declaration of Helsinki and approved by the Institutional Review Board of the institution; the requirement for written informed consent was waived owing to the retrospective design.

Clinical and radiographic assessments were performed preoperatively and postoperatively at routine intervals (within the first two postoperative weeks and at approximately 3, 6, 12 and 24 months). Radiographic decompression was assessed on the standardised early postoperative imaging obtained during the initial admission, whereas the analysis of clinical (VAS) outcomes was restricted to patients who had reached a minimum follow-up of 24 months. Of the 236 patients, 181 (76.7%) met this 24-month threshold at the time of analysis and constituted the clinical-outcome cohort; radiographic parameters were available for the full cohort.

Sex was included as a biological variable. Both male and female patients were enrolled, and outcomes were analysed for the whole cohort; the indirect decompression mechanism under study was not expected to differ by sex.

### 2.2. Surgical Technique

All procedures were performed by two neurosurgeons (J.-T.H. and J.-W.H.) under an identical protocol. With the patient in the right lateral decubitus position, a left-sided oblique pre-psoas (retroperitoneal) approach was used with a tubular retractor. After discectomy and endplate preparation, an interbody cage was inserted using an octagonal manoeuvre to obtain maximal endplate coverage and disc-height restoration. Intraoperative neuromonitoring was not used. Posterior instrumentation was added in the same setting—either one- or two-level percutaneous pedicle screw fixation (PPF) or open transpedicular screw fixation (TPSF)—without any laminectomy, facetectomy, foraminotomy, or other posterior decompression.

### 2.3. Clinical Assessment

Clinical outcomes were assessed using the visual analogue scale (VAS) for back and leg pain, recorded preoperatively and at the final follow-up. Perioperative variables—anaesthesia time, operative (skin-to-skin) time, estimated blood loss, postoperative drainage, transfusion, length of hospital stay, ambulatory recovery time and complications—were also recorded.

### 2.4. Radiographic Measurements

Radiographic parameters were measured on plain lateral radiographs and on sagittal and axial T2-weighted magnetic resonance imaging (MRI) using the electronic cursor of the picture archiving and communication system (PACS). Postoperative MRI was obtained during the initial postoperative admission to standardise the timing of the decompression assessment, as far as was feasible in a retrospective cohort. The following parameters were assessed:

(1) Disc height—measured on lateral radiographs by the Frobin method as the mean of the four corner distances, DH = (H1 + H2 + h1 + h2)/2 [7].
(2) Foraminal height—the maximum distance between the inferior margin of the pedicle of the superior vertebra and the superior margin of the pedicle of the inferior vertebra [8].
(3) Foraminal area—the cross-sectional area (mm^2^) of the neural foramen on sagittal T2 MRI [8].
(4) Spinal canal diameter—the antero-posterior diameter of the spinal canal at the mid-portion of the disc space on axial T2 MRI [9].
(5) Cross-sectional area (CSA) of the thecal sac—the cross-sectional area of the dural sac at the disc level on axial T2-weighted MRI [10].

All parameters were measured independently by two observers who were blinded to each other’s results, and the discrepancy between the two sets of measurements was recorded to assess inter-observer reliability. For each parameter, the extension (improvement) ratio was calculated as (postoperative − preoperative)/preoperative × 100%, and the relationship between the extension ratio and the corresponding preoperative value was examined. A predefined subgroup analysis at the L4–5 level compared segments with overt slippage (>Meyerding grade I) against segments with stenosis only (no slippage).

### 2.5. Statistical Analysis

Continuous variables are presented as mean ± standard deviation. The normality of the distributions was examined with the Shapiro–Wilk test. Paired preoperative–postoperative comparisons were made with the paired *t*-test or the Wilcoxon signed-rank test according to the distribution of the data, and between-group (slippage vs. stenosis) comparisons were made with the independent-samples *t*-test or the Mann–Whitney U test. The association between each extension ratio and the corresponding preoperative value was assessed with the Pearson correlation coefficient (or the Spearman coefficient for non-normally distributed variables). A two-sided *p*-value < 0.05 was considered statistically significant. Because the present analysis was primarily descriptive and univariate, multivariable modelling to identify independent predictors of successful indirect decompression was not undertaken.

## 3. Results

### 3.1. Baseline Characteristics

The cohort comprised 236 patients with a mean age of 59.1 ± 10.2 years; 110 (46.6%) were men and 126 (53.4%) were women. The leading diagnosis was spinal stenosis (117 patients, 49.6%), followed by spondylolisthesis (88, 37.2%) and herniated intervertebral disc (31, 13.1%). The mean preoperative VAS scores were 6.9 ± 2.6 for back pain and 7.6 ± 1.3 for leg pain, with a mean symptom duration of 23.6 ± 14.9 months. Four patients (1.7%) had undergone previous lumbar microdiscectomy. Baseline data are summarised in Table 1.

### 3.2. Operative and Perioperative Data

A single level was treated in 140 patients (59.3%) and two levels in 96 (40.7%), giving 331 operated segments. The most frequently treated level was L4–5 (145 patients, 61.4%), followed by L3–4 (130, 55.1%), L2–3 (51, 21.6%) and L1–2 (6, 2.5%). The mean anaesthesia and operative times were 354 ± 64 and 262 ± 72 min, respectively, with a mean estimated blood loss of 117.2 ± 44.3 mL and postoperative drainage of 83 ± 34 mL. Seven patients (2.9%) required transfusion. The mean length of stay was 5.6 ± 2.1 days and the mean ambulatory recovery time was 1.4 ± 0.6 days. Perioperative complications occurred in four patients (1.6%), including one retroperitoneal haematoma that required revision surgery (Table 2).

### 3.3. Clinical Outcomes

Clinical outcomes at a minimum of 24 months were available in 181 of the 236 patients (76.7%); the remaining 55 patients had either not yet reached 24 months of follow-up at the time of analysis or had incomplete final-visit scores. In the 181-patient outcome cohort, the VAS for back pain improved from 7.8 ± 2.1 preoperatively to 1.6 ± 1.8 at the final follow-up (*p* = 0.02), and the VAS for leg pain improved from 7.6 ± 1.3 to 2.0 ± 1.2 (*p* = 0.01). Six patients (2.5%) developed symptomatic residual neural compression that did not respond to the indirect decompression and who subsequently underwent additional posterior decompression. The potential for attrition bias introduced by the incomplete 24-month follow-up is considered in the Discussion.

### 3.4. Radiographic Outcomes

Substantial dimensional increments were observed in every radiographic parameter (Table 3). The mean disc height increased by 49.1%, foraminal height by 33.7%, foraminal area by 44.5%, spinal canal diameter by 37.4% and CSA of the thecal sac by 36.2%; all changes were statistically significant. The discrepancy between the two independent observers was small for the linear measurements (1.2–6.3%) and somewhat larger for the area measurements (12.5–14.3%). When the right and left neural foramina were compared, the foraminal height and area increased symmetrically on both sides, indicating a balanced bilateral decompressive effect.

In absolute terms, these percentage gains corresponded to mean increases of 5.3 mm in disc height (10.9 to 16.2 mm), 5.6 mm in foraminal height (16.5 to 22.1 mm), 40.7 mm^2^ in foraminal area (91.5 to 132.2 mm^2^), 3.4 mm in spinal canal diameter (8.8 to 12.2 mm) and 30.2 mm^2^ in thecal-sac CSA (83.6 to 113.8 mm^2^). These changes are clinically meaningful: the mean postoperative thecal-sac CSA rose above the approximately 100 mm^2^ value commonly associated with symptomatic central stenosis [10], and the radiographic enlargement was accompanied by significant relief of back and leg pain in the clinical-outcome cohort.

### 3.5. Correlation Between Extension Ratio and Preoperative Values

The extension ratios were inversely correlated with the corresponding preoperative values; that is, the more severely compromised a parameter was before surgery, the greater the proportional improvement after surgery. The correlation was strong and significant for disc height (r = −0.639, *p* < 0.001) and foraminal area (r = −0.545, *p* < 0.001), and modest but significant for foraminal height (r = −0.297, *p* = 0.040). The inverse correlations for spinal canal diameter (r = −0.136, *p* = 0.181) and CSA of the thecal sac (r = −0.190, *p* = 0.108) did not reach statistical significance (Table 4).

### 3.6. Slippage Versus Stenosis Subgroup at L4–5

At the L4–5 level, 42 segments with overt slippage (>Meyerding grade I) were compared with 36 segments with stenosis only. The slippage subgroup achieved greater extension ratios than the stenosis subgroup across all radiographic parameters—disc height (48.1% vs. 40.1%), foraminal height (33.8% vs. 27.4%), foraminal area (42.6% vs. 36.7%), canal diameter (38.2% vs. 33.1%) and CSA (34.4% vs. 32.6%)—a difference that reached statistical significance, indicating that segments with more severe preoperative deformity benefited most from indirect decompression (Table 5).

### 3.7. Illustrative Case

**Figure 1:**
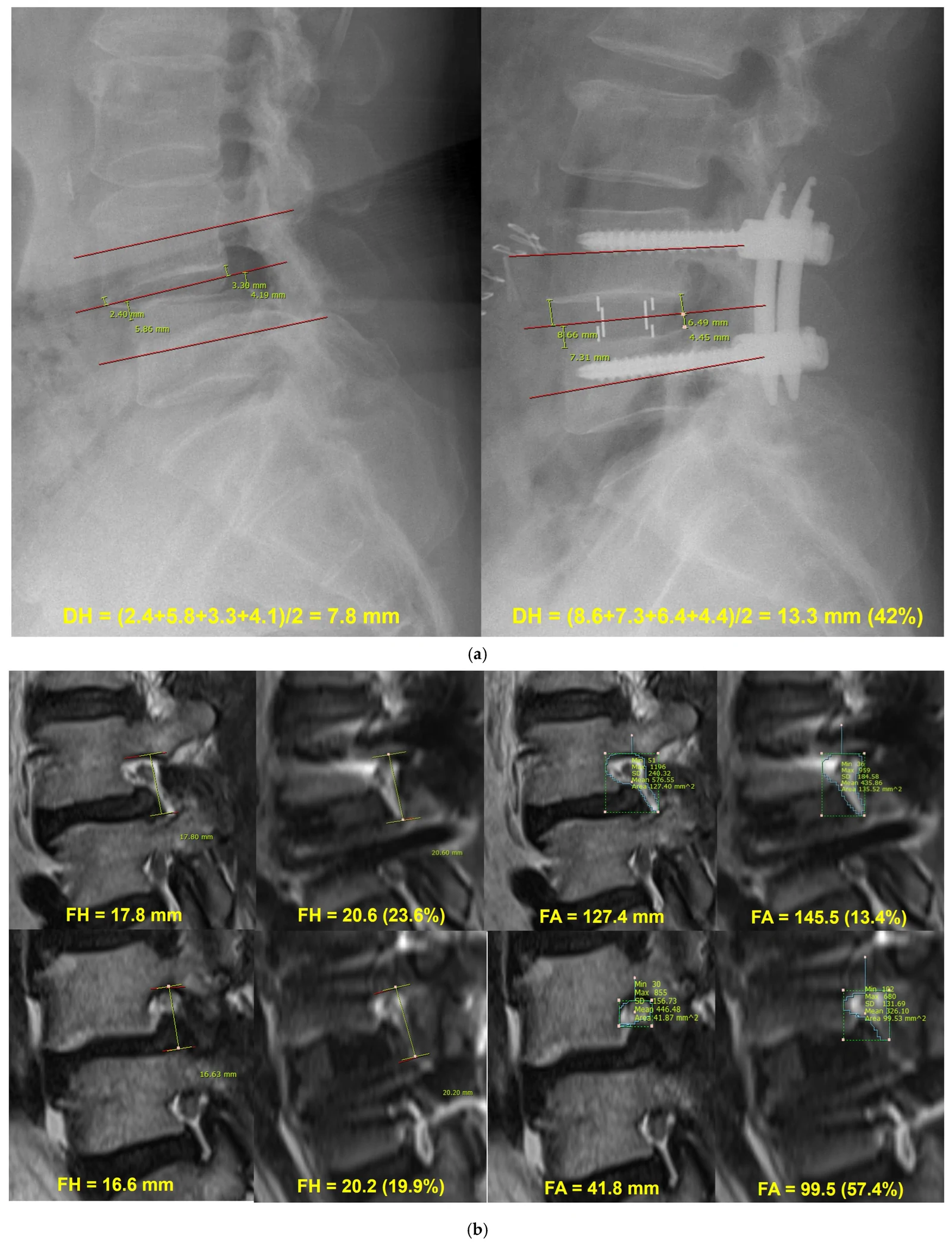
Preoperative and postoperative imaging of the illustrative case treated with MIS-OLIF without posterior decompression. (**a**) Preoperative (left) and postoperative (right) lateral radiographs showing restoration of disc height (DH) from 7.8 mm to 13.3 mm (+42%); (**b**) preoperative and postoperative sagittal T2-weighted MRI showing the corresponding increases in foraminal height (FH) and foraminal area (FA).

**Figure 2:**
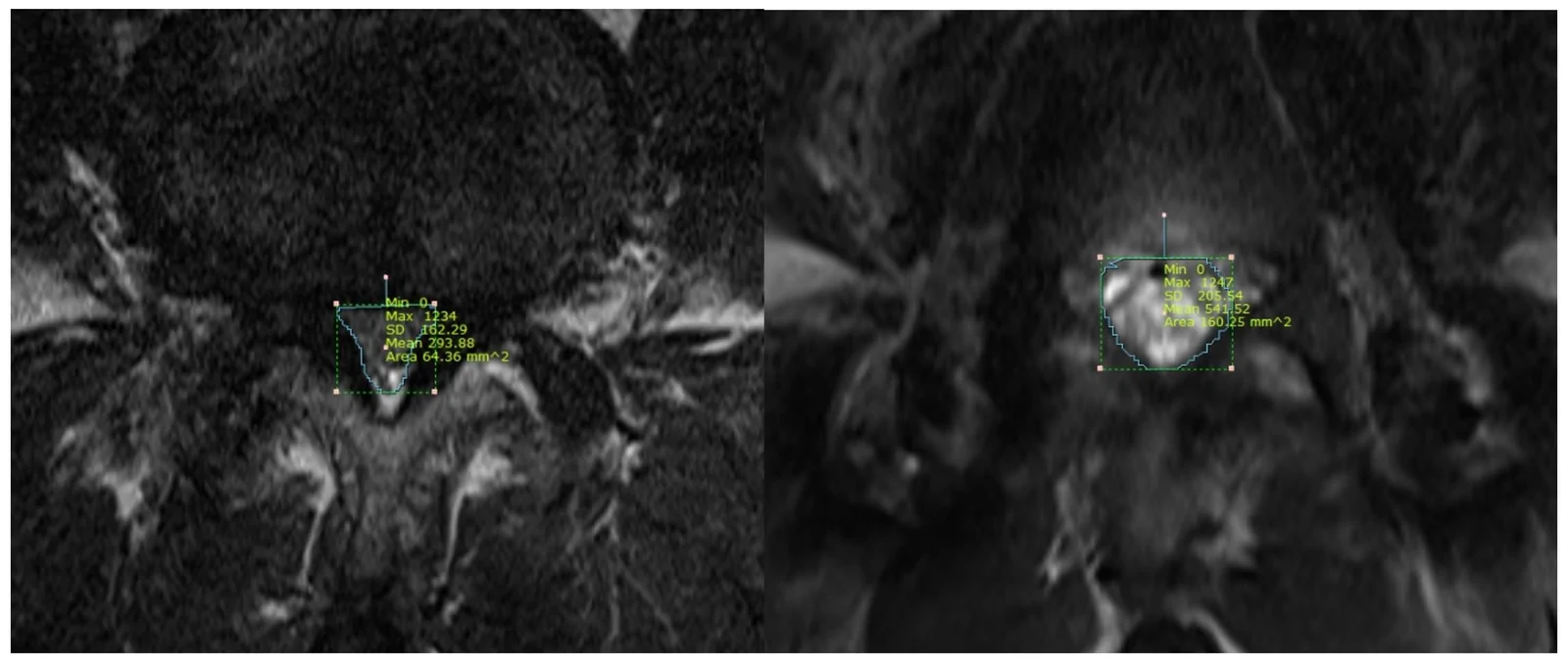
Preoperative and postoperative axial T2-weighted MRI of the illustrative case at the L4–5 disc level, showing enlargement of the spinal canal diameter and the cross-sectional area of the thecal sac.

A representative 62-year-old woman with L4–5 degenerative spondylolisthesis and neurogenic claudication underwent MIS-OLIF without posterior decompression (lateral stage 45 min, posterior fixation 42 min; estimated blood loss 220 mL). Indirect decompression restored the disc height from 7.8 to 13.3 mm and enlarged the spinal canal diameter (5.7 to 12.3 mm) and thecal-sac CSA (64.3 to 160.2 mm^2^), with resolution of symptoms (Figure 1 and Figure 2).

## 4. Discussion

To our knowledge, this is one of the largest consecutive, single-protocol series to quantify the indirect decompressive effect of MIS-OLIF performed strictly without any posterior decompression, and one of the few to link the magnitude of that decompression to the preoperative severity of each segment. Its principal contribution is therefore practical as well as mechanistic: by showing which segments respond most to ligamentotaxis, it offers spine surgeons a rational basis for selecting candidates for a posterior-sparing, minimally invasive strategy. In this cohort of 236 patients, MIS-OLIF without posterior decompression produced significant clinical improvement and substantial enlargement of every measured radiographic dimension of the neural foramen and spinal canal. The increases in foraminal area (44.5%) and thecal-sac CSA (36.2%), achieved without resecting any posterior osteoligamentous structure, support the contention that effective indirect decompression can be obtained through interbody distraction and ligamentotaxis alone in appropriately selected cases of degenerative pathology.

The proposed mechanism is twofold. First, restoration of disc height with a large, well-seated cage re-tensions the annulus fibrosus and the PLL; the taut PLL is thought to exert a ligamentotaxis effect that draws the bulging disc and posteriorly displaced soft tissue anteriorly, away from the dural sac. Second, distraction unbuckles the redundant, infolded ligamentum flavum and lifts the superior articular process, opening both the central canal and the neural foramen. Because these mechanisms were inferred from the imaging changes rather than measured directly, they should be regarded as the most plausible explanation for the canal enlargement observed here in the absence of any direct bony or ligamentous removal, rather than as a proven causal pathway. These findings are nonetheless concordant with earlier radiographic studies of lateral access fusion, which reported reliable increases in disc and foraminal dimensions and in central canal area after extreme lateral and oblique lateral interbody fusion [2,3].

A distinctive observation in our series is the inverse relationship between the magnitude of improvement and the preoperative value of each parameter. Segments that were most severely collapsed or stenotic before surgery exhibited the greatest proportional gains, an effect that was strong and significant for disc height and foraminal area. This is intuitive from a ligamentotaxis standpoint: a more collapsed disc space and a more lax, redundant PLL provide greater scope for height restoration and for the corrective pull on displaced tissue, whereas a segment with relatively preserved height has little room for further distraction. The corresponding subgroup analysis reinforced this pattern—segments with overt spondylolisthesis (>grade I) at L4–5 achieved larger extension ratios across all parameters than stenosis-only segments. Reduction in the slip during cage insertion adds a direct realignment component to the indirect decompression, which likely explains the superior radiographic response in the slippage subgroup, in keeping with previous radiographic reports of OLIF for degenerative spondylolisthesis [11].

Notably, the inverse correlations for central canal diameter and thecal-sac CSA, although present, did not reach statistical significance. This suggests that the central canal response to indirect decompression is less predictable than the foraminal response, which is restored more reliably through distraction and ligamentotaxis [12]; central stenosis is more often driven by fixed structural elements—facet hypertrophy, ossified or calcified ligamentum flavum, and bony lateral recess narrowing—that are not amenable to ligamentotaxis. This interpretation is consistent with the clinical course of the six patients (2.5%) who required subsequent posterior decompression. From a selection standpoint, the patients least likely to benefit from stand-alone indirect decompression are those with fixed bony lateral recess stenosis, congenital or bony central canal stenosis, a hypertrophied or ossified ligamentum flavum, severe facet arthropathy, bony (as opposed to height-dependent) foraminal stenosis, and high-grade spondylolisthesis. These features overlap closely with the reported radiographic predictors of failed indirect decompression [13] and with the preoperative factors shown to influence the success of immediate indirect decompression after OLIF [14]. Defining quantitative preoperative thresholds—for example, a critical bony canal dimension or degree of ligamentous ossification—that reliably separate responders from non-responders is an important next step; our univariate data indicate the direction of these relationships but are not sufficient to establish definitive cut-off values, which will require prospective, multivariable and receiver-operating-characteristic analyses.

The durability of indirect decompression also deserves emphasis. The initial enlargement of the foramen and canal can be eroded over time by cage subsidence, which settles the interbody space and may partially reverse the height restoration on which ligamentotaxis depends [15]; wider cages and attention to endplate preparation and bone quality mitigate this risk. Long-term considerations that were not captured in the present analysis include the eventual fusion rate, the maintenance of segmental and global sagittal balance, and the development of adjacent-segment disease, which remains a recognised late complication of lumbar arthrodesis and a driver of reoperation [16]. Because our follow-up focused on the perioperative period and on radiographic decompression, these outcomes could not be quantified here and are acknowledged as limitations; they should be primary endpoints of future work.

The clinical attractions of an indirect, posterior-sparing strategy are well illustrated by the perioperative profile of this cohort: modest blood loss (mean 117 mL), short ambulatory recovery (mean 1.4 days) and a low complication rate (1.6%), with preservation of the posterior tension band and the absence of laminectomy-related risks such as durotomy, epidural haematoma and postoperative fibrosis. The 2.5% rate of conversion to additional posterior decompression in the present series compares favourably with the failure and reoperation rates reported for indirect decompression when patient selection is not optimised [13,14], reinforcing the view that outcomes depend heavily on choosing segments whose compression is predominantly soft-tissue and height-dependent. The use of a pre-psoas oblique corridor, rather than a trans-psoas route, further reduced the dependence on intraoperative neuromonitoring and facilitated access to L4–5, the most frequently treated level in this series.

This study has several limitations. It is a retrospective, single-centre analysis without a concurrent control group undergoing direct posterior decompression, so the relative efficacy of the two strategies cannot be inferred. Clinical assessment relied on the VAS alone, without a validated disability or quality-of-life instrument such as the Oswestry Disability Index or SF-36, and neither fusion status, cage subsidence, adjacent-segment disease nor segmental sagittal balance was tracked. Clinical outcomes at 24 months were available for 181 of 236 patients (76.7%), and this attrition may introduce bias. The analysis was univariate; multivariable modelling to identify independent predictors of successful decompression, and formal confidence intervals and effect sizes for the observed changes, were not computed and should be added in future analyses of the complete dataset. Radiographic measurement, particularly of the foraminal and thecal-sac areas, is subject to inter-observer variability, as reflected in the larger discrepancy values for the area parameters, and although postoperative MRI was obtained during the initial admission the timing was not rigidly standardised. Finally, the indications applied here reflect the judgement of two experienced surgeons, which introduces selection bias and may limit generalisability. Prospective, comparative studies against direct decompression, with standardised imaging follow-up, validated functional outcome measures, longer-term assessment of fusion and adjacent-segment health, and quantitative selection thresholds, are warranted to confirm these findings.

## 5. Conclusions

MIS-OLIF without posterior decompression produced significant clinical improvement and significant enlargement of the neural foramen and spinal canal in patients with degenerative lumbar disease. Restoration of disc height reduced disc bulging, and ligamentotaxis of the posterior longitudinal ligament may have contributed to decompression of the spinal canal, with the greatest radiographic improvement seen in segments that were most severely degenerated or overtly slipped before surgery. Indirect decompression by ligamentotaxis therefore appears to be an effective, posterior-sparing treatment option for carefully selected patients, whereas a small subset with fixed structural compression will still require posterior decompression. Prospective, comparative studies with standardised imaging follow-up, validated functional outcome measures, longer follow-up and objective preoperative selection thresholds are needed to confirm these findings and to define the precise indications for, and durability of, the technique.

## Figures and Tables

**Table 1:** Baseline demographic and clinical characteristics (n = 236).

Characteristic	Value
Age, years	59.1 ± 10.2
Sex, male/female, n (%)	110 (46.6)/126 (53.4)
Diagnosis, n (%)	
Spinal stenosis	117 (49.6)
Spondylolisthesis	88 (37.2)
Herniated intervertebral disc	31 (13.1)
Preoperative VAS, back pain	6.9 ± 2.6
Preoperative VAS, leg pain	7.6 ± 1.3
Symptom duration, months	23.6 ± 14.9
Previous lumbar surgery, n (%)	4 (1.7)

VAS, visual analogue scale. Values are mean ± standard deviation unless otherwise indicated. All previous surgeries were microdiscectomy.

**Table 2:** Operative and perioperative data (n = 236; 331 operated segments).

Variable	Value
Levels treated, n (%)	
One level	140 (59.3)
Two levels	96 (40.7)
Operated segment, n (%) *	
L1–2	6 (2.5)
L2–3	51 (21.6)
L3–4	130 (55.1)
L4–5	145 (61.4)
Anaesthesia time, min	354 ± 64
Operative (skin-to-skin) time, min	262 ± 72
Estimated blood loss, mL	117.2 ± 44.3
Postoperative drainage, mL	83 ± 34
Transfusion, n (%)	7 (2.9)
Length of hospital stay, days	5.6 ± 2.1
Ambulatory recovery time, days	1.4 ± 0.6
Perioperative complication, n (%)	4 (1.6)

* Percentages are calculated against the number of patients; a patient could contribute more than one operated segment.

**Table 3:** Radiographic parameters before and after MIS-OLIF without posterior decompression.

Parameter	Preop	Postop	Δ (%)	Disc. (%) †	*p*-Value
Disc height (mm)	10.9 ± 4.9	16.2 ± 6.7	49.1	1.2	<0.01
Foraminal height (mm)	16.5 ± 5.2	22.1 ± 9.1	33.7	5.6	<0.05
Foraminal area (mm^2^)	91.5 ± 24.9	132.2 ± 47.4	44.5	14.3	<0.01
Spinal canal diameter (mm)	8.8 ± 2.9	12.2 ± 3.7	37.4	6.3	<0.05
CSA of thecal sac (mm^2^)	83.6 ± 32.1	113.8 ± 44.3	36.2	12.5	<0.05

Δ, extension ratio = (postop − preop)/preop × 100%; CSA, cross-sectional area. † Discrepancy between values measured independently by two observers. Values are mean ± standard deviation.

**Table 4:** Correlation between the extension ratio and the preoperative value for each parameter.

Comparison	Correlation Coefficient (r) ‡	*p*-Value
Δ Disc height vs. preoperative disc height	−0.639	<0.001
Δ Foraminal height vs. preoperative foraminal height	−0.297	0.040
Δ Foraminal area vs. preoperative foraminal area	−0.545	<0.001
Δ Canal diameter vs. preoperative canal diameter	−0.136	0.181
Δ CSA vs. preoperative CSA	−0.190	0.108

‡ Pearson correlation coefficient. CSA, cross-sectional area of the thecal sac.

**Table 5:** Radiographic outcomes in the slippage versus the stenosis subgroup at L4–5.

Parameter	Slippage Group (n = 42)			Stenosis Group (n = 36)		
	Preop	Postop	Δ%	Preop	Postop	Δ%
Disc height (mm)	10.9 ± 5.1	16.1 ± 6.2	48.1	10.9 ± 3.9	15.2 ± 4.2	40.1
Foraminal height (mm)	17.7 ± 4.8	23.6 ± 7.9	33.8	15.4 ± 3.7	19.6 ± 9.4	27.4
Foraminal area (mm^2^)	105.2 ± 22.1	150.1 ± 42.7	42.6	77.6 ± 14.2	106.7 ± 27.1	36.7
Canal diameter (mm)	5.7 ± 1.2	7.8 ± 3.9	38.2	12.8 ± 4.2	17.4 ± 4.1	33.1
CSA (mm^2^)	81.7 ± 23.2	109.8 ± 34.1	34.4	85.5 ± 27.7	113.4 ± 34.9	32.6

Δ%, extension ratio. CSA, cross-sectional area of the thecal sac. The lower preoperative canal diameter in the slippage group reflects narrowing produced by the vertebral slip.

## Data Availability

The data presented in this study are available on reasonable request from the corresponding author. The data are not publicly available owing to privacy restrictions.

## Abbreviations

The following abbreviations are used in this manuscript:

MIS-OLIF	Minimally invasive oblique lateral interbody fusion
PLL	Posterior longitudinal ligament
VAS	Visual analogue scale
ODI	Oswestry Disability Index
MRI	Magnetic resonance imaging
CSA	Cross-sectional area
PPF	Percutaneous pedicle screw fixation
TPSF	Transpedicular screw fixation
DLIF/XLIF	Direct/extreme lateral interbody fusion
